# How Can We Strengthen the Global Genetic Resources’ Conservation and Use System?

**DOI:** 10.3390/plants13050702

**Published:** 2024-03-01

**Authors:** Johannes M. M. Engels, Andreas W. Ebert

**Affiliations:** 1 Independent Researcher, 06062 Citta’ della Pieve, PG, Italy; 2 Independent Researcher, 73529 Schwäbisch Gmünd, Germany; ebert.andreas6@gmail.com

**Keywords:** ex situ conservation, global germplasm conservation and use systems, International Treaty on Plant Genetic Resources, Convention on Biological Diversity, Nagoya Protocol, governance of genetic resources, access and benefit sharing, genomics, phenomics, plant breeding

## Abstract

Genetic resources serve as the foundation of our food supply and are building blocks for the development of new crop varieties that support sustainable crop production in the face of climate change, as well as for the delivery of healthy diets to a continuously growing global population. With the encouragement of the FAO and with technical guidance and assistance from the International Board for Plant Genetic Resources (IBPGR), almost 2000 genebanks have been established worldwide for the ex situ conservation of genetic resources since the middle of the last century. The global genetic resources’ conservation and use system has evolved over several decades and presents apparent weaknesses, without a clear blueprint. Therefore, a Special Issue (SI) of *Plants* on ‘A Critical Review of the Current Approaches and Procedures of Plant Genetic Resources Conservation and Facilitating Use: Theory and Practice’ was initiated. This SI comprises 13 review and research papers that shed light on the history and the political dimensions of the global system; its current strengths, weaknesses, and limitations; and how the effectiveness and efficiency of the system could be improved to satisfy the germplasm users (plant breeders, researchers) and benefit consumers and society at large. This SI provides insight into new approaches and technical developments that have revolutionised ex situ conservation and the use of germplasm and related information. It also reflects on complementary conservation approaches (in situ, on-farm, home gardens) to ex situ genebanks, as well as how—through new forms of collaboration at national, regional, and global levels and through stronger links between public genebanks—synergies between the private breeding sector and botanic garden community could be achieved to strengthen the global conservation and use system. Special attention has also been given to the governance of genetic resources and access and benefit-sharing issues that increasingly hamper the needed access to a wide range of genetic resources that is essential for plant breeders to fulfil their mission.

## 1. Introduction

Targeted and considerable conservation efforts initially focused on threatened animals and subsequently on threatened ecosystems. The establishment and management of national parks and other forms of ‘in situ’ nature conservation were the mainstay conservation approach. It was only during the first half of the last century that crop genetic resources started to receive specific attention, often connected with plant breeding activities. A more ‘systematic’ ex situ conservation of plant genetic resources was possibly marked by the collection expeditions of Nikolai Iwanowitsch Vavilov, the publication of the results of his collection efforts, and the subsequent genetic diversity studies that he conducted in the 1920s/1930s [1,2,3,4]. This initial global and systematic approach was further strengthened during the so-called Green Revolution in the 1960s/1970s [5]. This resulted in more focused research on appropriate tools and methods, and a global coordination of collection and conservation efforts was undertaken by the FAO and the International Board for Plant Genetic Resources (IBPGR) [6]. Subsequently, many new institutional and national genebanks were created, in situ and on-farm conservation sites were established, and a policy and legal framework was developed and agreed upon [7,8]. Simultaneously, increasing attention was given to access and use aspects of Plant Genetic Resources for Food and Agriculture (PGRFA).

The resulting conservation and use framework was never purposely ‘designed’ for efficient and effective long-term conservation; it was rather the result of a spontaneous ‘process’ based on limited available scientific knowledge and undoubtedly influenced by political considerations [9]. Further influences resulted from the political debates and agreements such as the Convention on Biological Diversity (CBD) and, subsequently, as part of the coordinating efforts of the FAO Commission on Genetic Resources for Food and Agriculture in Rome, which were loaded with many issues, often unrelated to the subject of the effective and efficient (long-term) conservation and use of plant genetic resources, e.g., benefit sharing and property issues [10]. Since roughly the turn of the 20th century, many new technologies and scientific understandings have become available, including molecular genetics and genomics, phenomics, informatics and bioinformatics, as well as communication technologies. These technologies and the resulting scientific knowledge have revolutionised the possibilities of better understanding crop genetic diversity and improving conservation and have facilitated the use of PGRFA [11].

The developments mentioned above serve as the backdrop of this Special Issue on ‘A Critical Review of the Current Approaches and Procedures of Plant Genetic Resources Conservation and Facilitating Use: Theory and Practice’. It aimed to include descriptions of the current practices/state of the art of routine conservation operations, followed by a critical review of what could or should be done (in theory) considering the newly available technologies and scientific knowledge as well as the experiences made with the current system. The scope and focus of this Special Issue were on the ex situ conservation of plant agrobiodiversity. However, due attention was also expected for the broader issues and circumstances in which the conservation efforts and the global system are embedded. Whereas short- and medium-term conservation aspects are considered necessary, especially to facilitate the use of conserved PGRFA, the primary focus of this Special Issue intended to be on the long-term conservation efforts that are expected to be rational, effective, and efficient, including the related facilitation of use efforts. It was further expected that papers contributing to this Special Issue would include a section on moving from the current scenario into a more rational, efficient, and effective long-term conservation and facilitated use approach. Social, economic, and political considerations and developments were also expected to be addressed, where relevant, to ensure a widely agreeable and supported acceptance of the proposed way forward.

In the following section, the main ideas of the 13 papers that have been published as part of the Special Issue are briefly presented. The grouping of the papers was based on a logical approach. One paper that addresses policy issues regarding the availability of PGRFA under the Plant Treaty [12], published in the section ’Plant Genetic Resources’ of *Plants*, was originally also scheduled to be part of this Special Issue. In Section 3, key messages from the published papers are formulated with the primary aim of demonstrating how these contribute to strengthening the global PGRFA conservation and use system.

## 2. Highlights of the Papers Published in This Special Issue

There is no concept paper on which the global plant genetic resources’ conservation and use system was built. The system as we know it today is the result of experiences gained with targeted ex situ conservation efforts that were triggered by massive losses of genetic resources, particularly landraces. These losses were caused by the rapid spread of newly bred varieties of the major food crops in the 1960s, especially in tropical and subtropical countries as part of the Green Revolution. The global conservation and use system was also impacted by the political debates that ensued during the late 1980s. Increasingly, the decisions on how best to proceed with the conservation in a technical manner were based on targeted research results. The history of the development of the global system in the context of the political and legal framework and the main components it eventually entailed are addressed in the paper by Engels and Ebert [5]. In a second paper, Engels and Ebert [13] described the role of active and base collections and the importance of linking germplasm conservation with use. The authors reviewed the strengths and weaknesses of the current global system and made several recommendations on how the inherent weaknesses could be overcome and how improvements could be made.

With the encouragement of the FAO Commission on Genetic Resources for Food and Agriculture to establish genebanks for the storage of collected genetic resources and with technical guidance and assistance from the IBPGR, almost 2000 genebanks have been established worldwide since the middle of the last century. Because plant genetic resources, genebanks, and the genetic resources they contain can be threatened, Herbold and Engels [14] analysed the different types of risks that undermine the safety and security of the genebanks and their collections and suggested remedies for how such risks can be reduced. Another critical aspect of the efficiency and effectiveness of genebanks is the assessment of their quality performance. This is addressed in a paper by Lusty et al. [15] in which the authors describe a genebank quality management system (QMS) for the long-term conservation of genetic resources, initially adopted by the genebanks of the CGIAR in 2014, known as the ‘Genebank QMS’. The Genebank QMS is based on the FAO Genebank Standards, which defines performance targets, and adheres to international regulatory policies. It is implemented along the entire genebank operation, from germplasm acquisition to distribution, and relies on sound scientific practices that are regularly updated. The Genebank QMS provides a transparent, trusted framework for efficient operation, monitoring, auditing, and external review.

The development and application of new genomic tools and high-throughput phenotyping technologies, combined with the use of advanced information technologies to analyse the resulting vast datasets, are essential to facilitate and strengthen the effective and efficient conservation of PGRFA and their use in modern plant breeding. Volk et al. [11] provided several examples of the successful integration of genomic and phenomic approaches and demonstrated how vital access to high-quality and standardised data is for present and future PGRFA conservation and use efforts. They also indicate that advances in statistical prediction may change how germplasm characterisation data are used for further evaluation and breeding. Visioni et al. [16] provided an overview of the management and exploitation practices of barley genetic resources, predominantly illustrated with examples from the international genebank of ICARDA. They explore the relationship between genebanks and participatory plant breeding and offer insights into the diversity and utilisation of barley genetic resources. The authors highlight the importance of these genetic resources for boosting barley productivity, addressing climate change impacts, and meeting the growing food demands. They also emphasise the need for complementary genotypic and phenotypic evaluation of genebank collections to efficiently use the existing but vastly untapped biodiversity of barley genetic resources in future breeding programmes.

As is widely known, much of the world’s genetic diversity of domesticated crops can still be found in farmers’ fields as well as in gardens around homesteads or in garden plots in urban areas. The paper by Korpelainen [17] describes the importance of home gardens as an ‘ecosystem’ that harbours important and unique diversity that has sometimes developed over centuries, making a significant contribution to food and nutrition security at the local level. Home gardens have facilitated the adaptation and domestication of plants, including to extreme or specific ecological conditions, and have thus contributed to the diversification of cultivated plants. It is well known that genetic resources of public interest, not directly linked with the agricultural sector, are also conserved ex situ in genebanks. One, and possibly the most important example of such conservation efforts, is provided by botanic gardens that have increasingly established seed and field genebanks for the long-term conservation of plant genetic resources, including those of crop wild relatives and wild food plants. Unfortunately, the cooperation between the agricultural and botanic sectors is still relatively weak, and a paper by Breman and colleagues [18] from the Millennium Seed Bank at Kew Gardens, UK, intends to provide arguments and reasons to establish or strengthen such cooperation. The authors highlight the importance of networking and facilitating access to the conserved materials as well as the need to combine in situ and ex situ conservation approaches.

Whereas the initial focus of the ex situ conservation of PGRFA has been predominantly on the establishment and operation of ex situ conservation facilities, i.e., genebanks, over time, it was realised that effective and efficient conservation could only be achieved through adequate cooperation between genebanks at the national, regional, and global level. Taking into account the establishment of growing conservation activities of wild and cultivated plants, the increasing research efforts to improve conservation efforts and to facilitate use, as well as the increasing importance of coordinating the participation of national researchers in regional and global conservation programmes, the establishment of national conservation programmes and facilities was recognised. Against this backdrop, the paper of Begemann et al. [19] provides insight into the complexity of coordinating and governing such a national system in Germany, a federal state with active conservation, research, and use programmes for food and forestry plants, animals, and aquatic resources. A more specific case of collaboration between genebanks established by private plant breeding companies and publicly funded genebanks has been reviewed by Engels et al. [20]. The authors interviewed private plant breeders, assessed the published literature, and analysed specific existing cooperation arrangements to allow a more informed decision when seeking to strengthen such collaboration at the national, regional, and global level. The regional level represents yet another dimension of network coordination between the conservation of PGRFA and the facilitation of their use by genebanks and national programmes. The paper by van Hintum et al. [21] explored the establishment and operation of a decentralised regional virtual genebank in Europe, i.e., AEGIS, containing unique and important germplasm that has been designated by the respective national coordinators of the regional collaboration programme ECPGR. To further strengthen AEGIS, a system of certified genebanks with proper quality management, guaranteeing the long-term conservation of, and immediate access to, the conserved germplasm materials, is being proposed. Considering the current changes, challenges, and opportunities that impact the conservation and use of PGRFA, Lusty et al. [22] presented their views of an effective global long-term agrobiodiversity conservation system that promotes and facilitates the use of PGRFA. They argued that the rapid expansion of the applications and uses of modern genomic and phenomic technologies and approaches had a transformational impact on breeding, research, and the demand for certain genetic resources and associated data. Genebanks need to be responsive and must adapt to these changing conditions. These trends also provide important opportunities for genebanks to reorganise themselves and become more efficient individually and as a community. Ultimately, future challenges and opportunities will drive more demand for specific and well-documented genetic diversity and provide an important basis for genebanks to gear up.

Over the years, policy issues have gradually gained significant importance. At present, it is difficult to manage genebanks or breed new crop varieties without good knowledge of national, regional, and global policies and legal issues. Under the Convention on Biological Diversity (CBD) and its related Nagoya Protocol, access to PGR became increasingly restricted and cumbersome, resulting in a decrease in germplasm exchange, potentially threatening the future of plant breeding. After a critical review of current access and benefit-sharing regulations regarding PGRFA, Ebert et al. [23] recommended, among others, expanding the scope of the International Treaty on Plant Genetic Resources for Food and Agriculture (ITPGRFA) to include all PGRFA and making them and all related information accessible under a Standard Material Transfer Agreement (SMTA), combined with a subscription system or a seed sales tax, if necessary. Such a transparent, functional, and efficient system would erase legal uncertainties and minimise transaction costs for conservationists, curators, and users of genetic resources, thus aiding plant breeders in fulfilling their mission.

## 3. Key Messages

### 3.1. The Current Global Ex Situ Conservation and Use System—A Reflection on Its Inherent Weaknesses and Recommendations for Its Improvement

The current global conservation system has inherent weaknesses and limitations, partially due to its spontaneous creation out of a felt need by concerned scientists and visionaries and the subsequently required adjustments to the evolving political framework and changing realities. Because of its relatively easy and transparent access to PGRFA, the International Treaty, with its multilateral system (MLS) and standard material transfer agreement (SMTA) that stipulates benefit-sharing mechanisms for the use of germplasm, is the most significant policy instrument for PGRFA currently. As of 1 December 2023, the Treaty had 151 contracting parties, including the European Union as a member organisation [24]. However, the MLS is restricted to a list of 35 crops or crop gene pools and 29 grass and forage species that are listed in Annex 1. This is a significant limiting factor as, for example, only a small number of vegetable crops and neglected and underutilised species have been included in the list. Furthermore, commodity crops, like coffee, cacao, and tea, are excluded.

Vegetables and other minor, underutilised crops are known to play a major role in food and nutrition security [25]. Hence, their significant underrepresentation in the global system is a clear weakness. For many minor and underutilised crops and crop wild relatives (CWRs), there are still no comprehensive collections, and considerable gaps remain to be filled.

Apart from the threat to genetic resources in nature and/or farmers’ fields as well as in genebanks, genebanks themselves are exposed to a series of risks that may originate from natural hazards, such as earthquakes and storms, but also from political or financial issues. Herbold and Engels [14] undertook risk analyses of 80 important national and international genebanks regarding natural hazards and political and financial risks, and they concluded that there are large differences in the risk exposure of genebanks, making a location- and institution-specific risk assessment indispensable. Such risk assessments would help create more awareness at the local or national (political) level, hopefully resulting in the implementation of measures that mitigate the impact of risks, both for the genebank structures as well as for the safety of the collections.

Well-organised and comprehensive information management in genebanks is the basis for efficient and effective conservation and use. Unfortunately, information management and the online accessibility of accession-level data remain weak in many genebanks, especially at the national level. The rationalisation of collections requires comprehensive accession-level data and is an important step toward more cost-efficient and effective PGRFA conservation and use activities. These are just some weaknesses or limitations of the current global conservation and use system. For more details, please refer to [13]. After a detailed review of the current system, Engels and Ebert [13] recommended several measures that might contribute to a more rational, effective, and efficient long-term ex situ conservation system. Some of these measures include the following:

- Targeted collection for filling genetic and geographic gaps in current ex situ collections of major and particularly minor crops to reach an adequate representation of crop gene pools in ex situ collections. This would also be important to avoid the irreversible loss of genetic diversity due to severe genetic erosion in farmers’ fields and in nature.
- Rationalisation of germplasm collections through the determination of unique accessions that will form part of global base collections, and removal of the many duplicates within and among genebanks from the currently existing base collections.
- Strengthening existing and forging new collaborations among genebanks that maintain agricultural (crop) collections, including with the botanic garden community as well as with research institutes that hold collections. These are realistic mechanisms to increase efficiency and security in genebank and germplasm management, both at the global and national level. Furthermore, establishing stronger linkages with the plant science research community at large would be another step to facilitate coordination, foster collaboration, and facilitate the sharing of responsibilities. The community of public- and private-sector breeders represents a stakeholder group of the current global conservation efforts that has a significant interest in maintaining the adequate genetic diversity of our crops. Strengthening the collaboration with the private sector, especially with the plant breeding companies that operate private genebanks, has been identified as an underdeveloped building block of the global conservation and use system [20]. Strengthening such collaborations will contribute to the more efficient and effective long-term conservation of crop gene pools. This issue is discussed in more detail in Section 3.4.
- In the fields of molecular genetics and information technologies (including artificial intelligence), the world is seeing rapid technological advances. Further (adaptive) research is needed to fully exploit and apply these new technologies to the conservation and use of genetic resources and/or collaboration with specialised or more advanced institutions. Thus, more investments in conservation research and user-oriented supportive research are needed to optimise routine genebank processes and to facilitate conserving and delivering germplasm resources of high quality and in the right form as required by the users.
- Comprehensive, reliable, and easily available information on conserved accessions is a prerequisite to facilitating targeted and sustainable use of conserved genetic resources. Consequently, there is a clear need for a better and more comprehensive accession-level description of the genetic diversity of crop collections maintained in genebanks, including genomic, phenomic, and ecological data [11,13,22]. Furthermore, users require easy access to high-quality data on conserved germplasm and associated metadata.
- Legal certainty and easy and transparent access to conserved genetic resources are possibly the most fundamental requirement to enable and facilitate their use. There are legal frameworks (ITPGRFA, CBD, Nagoya Protocol) in place that regulate germplasm access and related benefit sharing. However, due to the fact that only a limited number of crops fall under the multilateral system of the ITPGRFA and each country is free to establish its own bilateral access rules under the Nagoya Protocol, users often find it difficult to undergo such a time-consuming, bureaucratic, and also costly process, especially when genetic resources from more than one country are needed and legal certainty is not guaranteed. To strengthen and simplify the legal and policy framework, it seems unavoidable to include all PGRFA in the MLS of the International Treaty (or to create another legal system that embraces all PGRFA) to facilitate easy access to germplasm, associated information, and corresponding benefit sharing in a transparent manner.
- A model for a functional and efficient global network of base and active collections is recommended, and a lean international organisation is proposed that assumes responsibilities for the global coordination, facilitation, and oversight of the various global crop gene pool base collection networks. Such a model could build on the existing genebanks of the CGIAR, the World Vegetable Center, and ICBA, as well as on a handful of strong national genebanks that form the core of the current global system on PGRFA.
- The political oversight over the proposed global model network of base collections should remain with the FAO and the Governing Body of the International Treaty.

In summary, the proposed measures include filling genetic and geographic gaps in current ex situ collections; determining unique accessions at the global level for long-term conservation in virtual base collections; intensifying existing international collaborations among genebanks and forging collaborations with the botanic garden community; increasing investment in conservation research and user-oriented supportive research; improving the accession-level description of the genetic diversity of crop collections; improving the legal and policy framework; and overseeing the proposed network of global base collections [13].

### 3.2. New Approaches and Developments Regarding Ex Situ Conservation and Facilitating Use

The awareness of quality management of plant genetic resources is widely accepted as important by genebank curators. However, the daily practice in genebanks is often very different, and many circumstances do exist that ‘undermine’ standardised procedures, agreed (genebank) standards to be met, etc. The paper of Lusty et al. [15] makes a convincing case for how modern quality management systems can be used to improve the overall performance of genebanks and to achieve much better results in conserving materials for the long-term in base collections for efficiency and effectiveness. The Global Crop Diversity Trust (Crop Trust) served as coordinator of the CGIAR Genebanks Research Program (2012–2016) and established a monitoring system for operations across all CGIAR genebanks. The system comprises five elements: (1) performance targets, (2) online reporting, (3) a genebank quality management system (QMS), (4) a system-level Standard Operation procedure (SOP) documentation audit, and (5) external review and validation. Genebank curators are recommended to take a close look at the quality performance system that has been put in place by the CGIAR genebanks and to see which aspects could be applied to their genebank. Meanwhile, the World Vegetable Center and the Centre for Pacific Crops and Trees (CePaCT) genebanks have also started operating under the above-mentioned QMS.

In the fields of molecular genetics, phenomics, and information technologies, the world is witnessing significant advances that are gradually being utilised by genebanks to enhance the effectiveness and efficiency of genebank operations and to facilitate the use of conserved germplasm [11,13,16,22]. It is now possible to collect phenomic and genomic data for genebank accessions or entire collections, which, with the help of appropriate analytical tools, can be directly used by plant breeders to guide the selection of accessions for target traits or specific environments [11]. For traits with complex or uncertain genetic control, genomic selection is being used. For the selection of variants across the whole genome, an intelligent algorithm is required, as well as a good training population for the algorithm to learn from [26]. Genomic prediction also requires high-throughput phenotyping to develop and validate the algorithms [27].

Genebanks must catch up with these new technological developments to improve genebank operations and to accommodate the changing needs of breeders and researchers. There is a growing need for comprehensive online searchable repositories of information on genetic resources, as users increasingly require access to digital information associated with accessions, i.e., a shift to ‘digital genebanks’ [15,28]. An example in this direction is the AGENT project (Access to Genetic Resources and Digitisation of Plant Genetic Resources). AGENT aims to support the exploration of the untapped potential of the vast genetic resources stored in genebanks worldwide by leveraging FAIR (Findable, Accessible, Interoperable, and Reusable) international data standards and open digital infrastructure, thus facilitating germplasm use for breeding and research [16].

### 3.3. Other Forms of Conservation That Complement the Current Long-Term Conservation System

Home gardens may contain unique and rare, locally evolved or developed genetic diversity, as they harbour broad species and genetic diversity, including the wild relatives of our crops, and they can be found in almost any ecological condition that the inhabited world possesses. This makes this specific ‘ecosystem’ very interesting and relevant for the conservation of plant genetic resources as well as for the use of the frequently unique genotypes of a given crop or species that are being cultivated by smallholders. It is necessary to establish strong and effective linkages between home garden conservation efforts and the established ex situ conservation system at the local and national levels to ensure the safety of the in situ conserved materials and to facilitate their wider use [17].

Landraces still play a major role in crop cultivation and may reach up to 70% of cultivated areas, as has been shown in the case of barley [16]. Efforts by ICARDA have shown that the yield of dryland landraces can be significantly improved through seed cleaning and treatment against seed-borne diseases and through farmer-participatory selection, thus providing incentives to farmers for continued cultivation of such landraces, also as a form of on-farm conservation [29].

While ex situ conservation is a static process, on-farm conservation allows the manifestation of evolutionary processes in genetic resources and may lead to crop improvements and adaptation to changing climatic conditions over time. In this context, Evolutionary Participatory Breeding (EPB) is an exciting approach. It encompasses the planting of mixtures of diverse genotypes of the same crop in farmers’ fields. This mixture may consist of early segregating generations that maximise allelic diversity for specific traits of interest. Over successive crop cycles in the same environment, the mixed population will gradually evolve and adapt to the specific environmental conditions. Genotypes that are more adapted to that environment will progressively become more dominant, ensuring resilience and long-term adaptation [30].

As stated by Engels and Ebert [5], ex situ and in situ conservation should be combined to achieve long-term security and cost-effectiveness of PGRFA conservation. In this context, the evolving concept of trans situ conservation is worth mentioning, which, in the case of crop wild relatives, dynamically integrates multiple in situ and ex situ measures, from conservation to research to education, comprising local and global scales [31].

The conservation of predominantly wild plant genetic resources in botanic gardens takes place in a largely independent evolved global conservation system from the genebank system that is more crop-genetic-resource-oriented. Considering the fact that the genetic resources that are maintained in botanic gardens include many crop wild relatives as well as locally grown or collected, edible or otherwise useful species, it appears highly relevant to establish linkages and strengthen the cooperation between crop genebanks and the community of botanic gardens. Moreover, the skill sets found within botanic gardens and agricultural genebanks complement each other and enable the development of integrated conservation approaches. The botanic garden community is highlighting the importance of networks and is willing to provide access to data and plant material [18]. Stronger linkages and cooperation between crop genebanks and the botanic garden community would be an important step toward a more effective and efficient global conservation system.

### 3.4. National, Regional, and Global Efforts and Strategies for the Improvement in the Current Conservation and Use System

The coordination of conservation efforts at the national level can be regarded as an essential building block of the global conservation system. Many of the current global system elements are based on (voluntary) contributions made by individual sovereign states to frameworks such as the CBD and the International Treaty. The authors have experienced the centrally coordinated German national PGRFA conservation and use system as one of the more comprehensive, efficient, and strategic approaches worldwide. It is participatory, inclusive, dynamic, and forward-looking with information management at the heart of the coordination efforts [19].

As plant genetic resources follow natural distribution patterns and not political borders, it is of crucial importance that close collaboration between neighbouring countries is a key prerequisite for the effective and efficient conservation (and use) of individual crop gene pools (or parts thereof). Thus, the regional coordination and collaboration of PGRFA (as well as of non-PGRFA) are significant to harnessing existing strengths, infrastructure, and knowledge. The European Cooperative Programme for Plant Genetic Resources (ECPGR) has tried to follow this paradigm by creating a decentralised virtual genebank, abbreviated as AEGIS (A European Genebank Integrated System). AEGIS aims to establish a European Collection of unique and important accessions maintained in various genebanks scattered over Europe that adhere to the AEGIS concept and principles, thus reducing costs due to reducing redundancy in the numerous national and institutional genebanks across Europe [21]. As AEGIS currently depends on funding from national authorities, it is far from perfect. Additional strategic funding from the European Union is required to truly set up a system of AEGIS-certified genebanks, in which the quality and continuity of conservation of, and access to, PGRFA could be guaranteed [21]. The AEGIS approach provides an excellent example of how the conservation and use facilitation of PGRFA can contribute to a more rational and efficient approach embedded in a regional governance and financial structure.

At the regional and global level, the CGIAR genebanks safeguard some of the largest and most widely used collections of crop diversity, critical to attaining the UN’s Sustainable Development Goals (SDG) to end hunger and improve food and nutrition security [15]. Most CGIAR genebanks are strategically located in centres of crop diversity, therefore representing the rich genetic diversity of primary crop gene pools. Based on collection missions, donations, and genetic materials generated by breeders and researchers, the CGIAR collections have grown over the last five to six decades and harbour a rich diversity of landraces, heritage varieties, crop wild relatives, improved varieties, and breeding or research materials for specific mandate crops. The World Vegetable Center (WorldVeg), loosely aligned with the CGIAR, complements the CGIAR crop collections with a considerable diversity of vegetable genetic resources [25]). Through global and regional crop networks and collaborative projects, the CGIAR and WorldVeg have established close links with national PGRFA conservation and use programmes, thereby facilitating the efficient long-term conservation, use, and exchange of PGRFA. The efficient long-term conservation and distribution programmes established by the CGIAR and WorldVeg and their crop-specific breeding programmes serve as a model for the efficient and effective global PGRFA conservation and facilitating use efforts.

The Crop Trust served as coordinator of the CGIAR Genebanks Research Program (2012–2016) and put in place a QMS monitoring system for operations across all CGIAR genebanks. Details are provided in Section 3.2. Apart from the CGIAR genebanks, the Crop Trust also supports selected national genebanks in the Global South to safeguard the long-term conservation of critical genetic resources for food and nutrition security.

Private-sector breeders and curators of public national genebanks might have very different goals and objectives; however, genetic diversity is fundamental to both sectors. Therefore, any attempt to establish new or improve existing collaborations between the two sectors is expected to strengthen the global system. The often-existing mistrust in each other needs to be overcome through dialogue and communication to identify areas and activities of common interest.

Engels et al. [20] reported convincing examples of a close collaboration between public genebanks and private-sector breeders on the conservation of genetic resources. These examples include the Centre for Genetic Resources (CGN) in the Netherlands, the World Vegetable Center, and East-West Seed International. Over several decades, the CGN developed a fruitful collaboration with the vivid breeding industry in the Netherlands in areas that include regeneration, joint phenotyping and screening of accessions, and the funding of collection trips, including the benefit-sharing component. WorldVeg concluded agreements with private-sector companies to regenerate original accessions in order to make them available to users worldwide.

To accelerate the development and dissemination of elite vegetable crop materials, WorldVeg also concluded breeding consortia in Asia and Africa. East-West Seed International provides in-kind support to national and other domestic genebanks in the Philippines and Indonesia and also collaborates with the CGN in regenerating germplasm materials. These examples demonstrate that collaborative arrangements between (inter)national public genebanks and vegetable breeding companies, often coordinated by the respective seed associations, contribute significantly to germplasm collection, conservation, documentation, and their sustainable use, thus making a valuable contribution to the global system.

### 3.5. Governance and ABS Issues

Along with the evolution of the global conservation system, the ‘concept’ of genetic resources has evolved as well. Initially, the focus was strongly on the use of germplasm as the raw material for plant breeding, a resource that was freely available and considered a ‘common heritage’ of humankind. The term germplasm gradually started to also embrace the associated knowledge as well as information derived from germplasm through basic and applied research, and breeding. Simultaneously, the status and recognition of the role of cultivators/custodians of the genetic resource became part of the picture, and the concept of benefit sharing resulting from the use of the acquired resources was added to the legal arrangements to obtain these. Gradually, not only did the reproductive organ of the genetic resource become a legal ‘substance’, but also its genetic components as well as digital sequence information derived from germplasm. This has made arrangements to share this essential resource with others quite complex, resembling a ‘legal jungle’ [13,23].

The Convention on Biological Diversity (CBD), the International Treaty on Plant Genetic Resources for Food and Agriculture (ITPGRFA), and the Nagoya Protocol are relatively recent international agreements that recognise the sovereign rights of countries over their genetic resources. Under the CBD/Nagoya Protocol, countries are free to establish specific national legislations that regulate germplasm access and benefit sharing to be negotiated bilaterally. The need to negotiate bilateral agreements, often with several countries to access specific genetic resources, turned out to be a cumbersome, highly bureaucratic, time-consuming, and costly effort, resulting in a decrease in or even a cessation of germplasm exchange. The ITPGRFA attempted to ease this situation by establishing a globally harmonised multilateral system (MLS). Unfortunately, the MLS is (still) restricted to a limited number of food and forage crops, with very few vegetable crops [23].

On the other hand, crop improvement depends on access to (agro)biodiversity to source new genetic variations for breeding. Fair, transparent, and non-bureaucratic rules and regulations that provide legal certainty concerning the access to and use of germplasm in breeding and research are, therefore, a predisposition for food and nutrition security. Germplasm users need to have clarity on whether the ITPGRFA, the CBD/Nagoya Protocol, or any other ABS tool apply. Furthermore, adjustments to the current texts of national legal instruments regarding ABS regulation under the Nagoya Protocol (preferably in a common universal language) are needed to ensure legal certainty and strengthen access to genetic resources. According to Ebert et al. [23], expanding the scope of the ITPGRFA to include all PGRFA, as well as related organisms like pathogens and pests, and making these genetic resources and all related information accessible under a Standard Material Transfer Agreement (SMTA) would greatly benefit the use of new germplasm in breeding and lead to the creation of improved varieties that can cope with climate change challenges and will contribute to more sustainable forms of agriculture. To facilitate benefit-sharing arrangements, the SMTA could stipulate a subscription system or a seed sales tax. Such a transparent, functional, and efficient system would erase legal uncertainties and minimise transaction costs for conservers, curators, and users of genetic resources, thus aiding plant breeders in fulfilling their mission.

### 3.6. Concluding Remarks

Genebanks need to adjust and embrace new technologies in the fields of molecular genetics, phenomics, and information technologies to improve the effectiveness and efficiency of genebank operations and to meet the evolving needs of users, both in terms of genetic resources and associated data, more accurately and efficiently than they do today [22]. Given the uncertainties with climate change, the need to develop climate-smart and -resilient crop varieties for sustainable crop production, and the need to feed the still-growing population with healthy food, plant breeders depend on the easy and non-complicated availability of a wide range of genetic diversity. The form in which this diversity is needed might change and breeders might ask for larger amounts of associated data. Genebank materials will be needed for gene discovery studies and for the identification of functional variants.

With the progress in technological advancements, adjustments to the above-mentioned international agreements are mandatory to secure and facilitate germplasm exchange. ABS mechanisms need to become transparent and easy to implement and adhere to, they need to provide legal certainty, and they need to have low transaction costs, thus benefiting providers and users of germplasm.

To enhance efficiency and reduce redundancy in crop collections and costs, a model of a functional and efficient global network of base and active collections, similar to the AEGIS concept in Europe, has been recommended [13]. A lean international organisation could assume responsibilities for the global coordination, facilitation, and oversight of the various global crop gene pool base collection networks. The Guest Editors of this SI hope that the results of this review inject new ideas into the ongoing discussions at the level of the Governing Body of the ITPGRFA and other fora and contribute to the needed reform of the global genetic resources’ conservation and use system.
